# An in vitro comparative study of the effects of soy milk and coconut milk on the microhardness of primary molar enamel after an acidic challenge

**DOI:** 10.1186/s12903-026-09193-7

**Published:** 2026-07-22

**Authors:** Alireza Hasanalizadeh, Hajar Attarzadeh Jouzdani, Parvin Mirzakoucheki Boroujeni, Yasmina Aalizadeh

**Affiliations:** 1 Department of Pediatric Dentistry, School of Dentistry, Islamic Azad University (Isfahan) Branch, Isfahan, Iran; 2 Department of Operative Dentistry, School of Dentistry, Islamic Azad University (Isfahan) Branch, Isfahan, Iran

**Keywords:** Enamel microhardness, Primary molar, Coconut milk, Soy milk, Acidic challenge, Artificial saliva

## Abstract

**Objectives:**

This in vitro study aimed to compare the effects of soy milk and coconut milk on primary molar enamel microhardness after an acidic challenge.

**Materials and methods:**

Microhardness of 30 primary molar enamel blocks was measured using a Vickers tester. For the acidic challenge, they were immersed in citric acid with a pH of 3 for 25 h, and then their microhardness was measured again. The specimens were then randomly assigned to 3 groups (*n* = 10) for immersion in either soy milk, coconut milk, or artificial saliva (control) 3 times a day, each time for 5 min, for 7 days. Subsequently, they underwent microhardness test again. Data were analyzed by ANOVA, independent t-test, and LSD test (alpha = 0.05).

**Results:**

The enamel microhardness significantly increased after immersion in coconut milk compared with the value after acidic challenge within the same group (*P* = 0.035). However, the difference in microhardness was not significant between the coconut milk and artificial saliva groups after the intervention (*P* = 0.178). No significant microhardness change occurred after soy milk immersion (*P* = 0.121), and no difference was found between soy milk and artificial saliva groups (*P* = 0.289).

**Conclusion:**

Under in vitro conditions, coconut milk significantly increased enamel microhardness after an acidic challenge, while soy milk showed a non-significant increase. Neither plant milk demonstrated a significant effect on microhardness compared with artificial saliva.

## Introduction

Tooth enamel is one of the most important dental structures in terms of both function and esthetics. Primary enamel reflects the metabolic and physiological events that occur during the intrauterine period and the first year of life [1]. Dental caries is the most common chronic disease worldwide, adversely affecting patients’ quality of life and increasing healthcare costs. According to epidemiological data, 60% to 90% of school-aged children in most industrialized countries have at least one carious tooth [2, 3].

Erosion is the most common cause of tooth wear. It is defined as irreversible destruction of the tooth structure due to a chemical process with no bacterial involvement. Acid exposure is usually the main cause of dental erosion. The acidic environment can be caused by extrinsic factors such as consumption of acidic foods, drinks, and medications, or intrinsic factors such as exposure of teeth to stomach acid due to vomiting. The main factors causing erosion in children include consumption of acidic drinks such as fruit juices that contain citric acid (e.g., apple juice and orange juice) and non-alcoholic carbonated beverages, esophageal reflux, or swimming in pools that contain non-standard concentrations of chlorine [4, 5].

Remineralization is defined as infusion of calcium and phosphate ions into the enamel defect to remineralize the subsurface layer. Such minerals are abundantly found in milk and dairy products [6]. The demand for plant-based milk alternatives is increasing due to various reasons including lactose intolerance and milk allergy, consumer concerns about the presence of hormones and cholesterol of cow milk, ethical controversies over animal use, environmental issues, lifestyle changes towards plant-based foods, and the health-promoting properties of these products [7, 8].

Soybean is a unique food source rich in proteins. They contain 35% to 45% protein and 20% fat, making them an important source of protein, especially for vegetarians [9]. Coconut milk is extracted from the white flesh of grated coconuts, and is rich in saturated fats [9]. It has been demonstrated that consumption of coconut milk can increase high-density lipoprotein which helps reduce harmful low-density lipoprotein in the bloodstream, due to its lauric acid content. Coconut milk is also a good source of minerals, including calcium, phosphorus, and potassium, and can aid in enamel remineralization [9]. Coconut milk is high in fat, fiber, and essential nutrients like iron, calcium, magnesium, and vitamins C and E, making it a rich thickener for food products. Consumption of coconut milk has numerous health benefits due to its anti-cancer, antimicrobial, antibacterial, and antiviral properties. It also contains lauric acid, which is a saturated fat found in breast milk and helps in brain development [10, 11]. Considering the significance of retaining primary teeth until their natural exfoliation, and the existing controversy regarding the effects of plant-based milks, this study aimed to assess the effects of soy milk and coconut milk on primary molar enamel microhardness after an acidic challenge. The first and second null hypotheses of the study were that coconut milk and soy milk would have no significant effect on primary enamel microhardness. The third null hypothesis of the study was that no significant difference would be found between the soy milk and coconut milk with the control group regarding the mean change in microhardness. Despite the increasing consumption of plant-based milks, evidence regarding their direct effects on primary enamel microhardness following acid challenges remains conflicting and sparse. Most previous studies [14–19] have focused on cow milk, used water as a control rather than artificial saliva which provides a better simulation of the oral environment, or did not specifically examine primary teeth. Furthermore, while some studies suggest cariogenic potential due to free sugars, others report remineralizing effects. This study shows the specific gap regarding the isolated effects of soy milk and coconut milk on enamel surface microhardness, without the confounding presence of bacterial biofilm or salivary pellicle, as a preliminary mechanical assessment.

Post-hoc comparison between plant milk groups: A direct comparison between the soy milk and coconut milk groups regarding the mean change in microhardness after remineralization (compared to post-demineralization) showed no statistically significant difference (mean difference 21.3, 95% CI -38.7 to 81.3, *P* = 0.412, independent t-test), indicating that neither milk was superior to the other under the tested conditions.

## Materials and methods

This experimental study used 30 extracted primary molars under ethical approval (IR.IAU.KHUISF.REC.1402.176). The parents consented to the use of extracted teeth of their children for research purposes. This study was conducted from September 2023 to February 2024 at the Dental Materials Research Laboratory of the Department of Pediatric Dentistry, IAU, Khorasgan Branch, Iran.

### Sample size

Based on Widanti et al. [12], sample size was calculated as 8 per group using two-way ANOVA (α = 0.05, power = 80%). Calculations used mean microhardness (438.87, 415.17) and standard deviations (22.44, 2.9) for test and control groups.

1 $$\:n=\frac{{\left({z}_{1-\alpha\:/2}+{z}_{1-\beta\:}\right)}^{2}({\sigma\:}_{1}^{2}+{\sigma\:}_{2}^{2})}{{({\mu\:}_{1}-{\mu\:}_{2})}^{2}}=\frac{(1.96+0.84{)}^{2}({22.44}^{2}+{2.9}^{2})}{{(438.87-415.17)}^{2}}=7.14$$

### Specimen preparation

The collected teeth were rinsed under running water and stored in distilled water in a glass container for 2 weeks [13]. The collected teeth were primary mandibular first and second molars and primary maxillary first and second molars, all with intact buccal or lingual enamel surfaces. Teeth with restorations, cracks, fluorosis, hypoplasia, or visible caries were excluded. The distilled water was refreshed once a week. The crowns were then separated from the roots by a cutting machine, rinsed, and dried. Enamel blocks measuring 4 × 4 × 2 mm were cut out of the tooth crowns by the cutting machine [4]. Enamel blocks with caries, discoloration, erosion, abrasion, or cracks were excluded. To ensure soundness of the blocks, they were inspected under a light microscope (Primo Star, Zeiss, Germany) at x40 magnification. Selected blocks were embedded in auto-polymerizing acrylic resin. Written informed consent was obtained from parents or guardians for using extracted primary teeth from routine dental procedures unrelated to this study. Microhardness testing was selected as a well-established, quantitative, and reproducible method for assessing surface mineral changes in enamel following demineralization and remineralization protocols. While complementary analyses such as scanning electron microscopy (SEM), energy-dispersive X-ray spectroscopy for Ca/P ratio, or transverse microradiography would provide additional information on subsurface lesion changes and true remineralization, microhardness testing remains an acceptable initial screening tool for comparative studies of this nature.

### Baseline microhardness testing

The enamel microhardness was initially measured by a Vickers hardness tester (Universal One; Koopa, Iran). For this purpose, 5 kg load was applied to the specimen surface for 10 s. In this study, each specimen and also three indentations were fabricated in various positions on the enamel surface, and the medium amount was declared for analysis. The distance between each indentation was standardized to at least 200 μm to avoid interference between adjacent indentations, and the distance from the indentation to the specimen edge was maintained at least 100 μm, in accordance with Vickers hardness testing standards. The microhardness (kgf/mm^2^) was reported in kilogram force per square millimeters.

### Acidic challenge and remineralization

The specimens were immersed in 20 mL of citric acid with a pH of 3 for 25 h. The solution was refreshed every 5 h [14]. Although a continuous 25-hour acid challenge at pH 3 is more aggressive than physiological conditions, this protocol was selected based on previous studies to create measurable and reproducible demineralization in primary enamel within a feasible experimental timeframe [15–20]. This limitation is explicitly acknowledged in the Discussion. Next, microhardness was measured for the second time. The specimens were then randomly assigned to three groups (*n* = 10). The specimens in the control group were immersed in 20 mL of artificial saliva. The specimens in the second group were immersed in 20 mL of soy milk (Mandasoy, Iran, Lot #MS1402-08, pH 6.9 ± 0.1, composition per 100 mL: protein 3.5 g, fat 2 g, carbohydrate 2.8 g, calcium 120 mg, phosphorus 90 mg, no added sugars). The specimens in the third group were immersed in 20 mL of coconut milk (Soul Food, Iran, Lot #SF1402-11, pH 6.7 ± 0.1, composition per 100 mL: fat 17 g, protein 2 g, carbohydrate 2.5 g, calcium 18 mg, phosphorus 22 mg, no added sugars). Both milks were stored refrigerated at 4 °C and used within 48 h of opening.

The specimens were immersed in 25 mL of the respective solutions three times a day, each time for 5 min, for a total of 7 days. In-between the immersions, the specimens were rinsed with distilled water, dried, and stored in artificial saliva (Chekad Sanaat, Iran) containing Na+, K+, Mg^2+^, Ca^2+^, Cl^−^, CO_3_^−^. P, and SO_4_^2−^ [12]. The artificial saliva (Chekad Sanaat, Iran) had a pH of 6.8 ± 0.2 and contained Na⁺, K⁺, Mg²⁺, Ca²⁺, Cl⁻, CO₃²⁻, P, and SO₄²⁻. The artificial saliva was refreshed every 24 h. After remineralization, the microhardness was measured for the third time, using the same method [15].

Specifically, each day for 7 consecutive days, specimens were immersed in the assigned solution (soy milk, coconut milk, or artificial saliva) for three separate sessions (morning, midday, evening) of 5 min each, for a total of 15 min per day. Between these immersion sessions, specimens were rinsed with 10 mL of distilled water for 10 s, gently dried with absorbent paper, and then stored in 20 mL of fresh artificial saliva at 37 °C. The artificial saliva was completely refreshed every 24 h. This cycle was repeated for 7 consecutive days.

The specimens were tested on their natural buccal or lingual enamel surfaces without grinding or polishing. Primary enamel naturally has a relatively smooth outer surface, and all specimens were inspected under light microscopy at x40 magnification to exclude those with surface defects, cracks, or irregularities. While polishing is standard in many microhardness studies, we deliberately chose not to polish to preserve the natural enamel surface, as polishing alters the outermost layer and may affect the response to demineralization. All specimens were handled identically, so comparisons between groups remain valid.

### Statistical analysis

Data normality was confirmed by Shapiro-Wilk test, and variance homogeneity by Levene and Mauchly’s tests (*P* > 0.05). ANOVA, independent t-test, and LSD test were used (SPSS 26, α = 0.05). Baseline microhardness showed no significant difference across groups (*P* = 0.374), confirming comparability.

## Results

In this study, Table 1 indicates descriptive statistics regarding enamel microhardness in the three groups at different time points. Enamel microhardness significantly decreased after the acidic challenge compared with baseline (*P* = 0.016).

**Table 1:** Mean (SD) enamel microhardness (kgf/mm²) in the three groups at different time points

Group	Baseline	After demineralization	After remineralization
Soy milk	173.2 (50.6)	147.4 (52.3)	213.6 (84.9)
Coconut milk	200.2 (49.6)	155.8 (50.6)	243.3 (96.7)
Control (artificial saliva)	214.2 (83.1)	177.5 (84.4)	197.4 (48.7)

### Within-group comparisons

ANOVA revealed a significant difference in the mean microhardness values in the coconut milk group at different time points (*P* = 0.0350). So, pairwise comparisons were done using the LSD test (Table 2), and the results showed that microhardness was significantly higher after remineralization than after demineralization (*P* = 0.044). No other significant differences were found within the coconut milk group (*P* > 0.05). Within-group comparison of microhardness at different time points revealed no significant difference in the soy milk (*P* = 0.121) or the control (*P* = 0.526) groups.

**Table 2:** Within-group comparison of enamel microhardness at different time points in the coconut milk group using the LSD test

Time (I)	Time (J)	Mean difference	Std. deviation	*P* value
Baseline	After demineralization	44.400	25.038	0.110
	After remineralization	+ 43.100	21.869	0.080
After demineralization	After remineralization	+ 87.500	40.476	0.044

### Between-group comparisons

The mean change in microhardness after demineralization compared with baseline (*P* = 0.825) and after remineralization compared with after demineralization (*P* = 0.289) was not significantly different in comparison of soy milk and control groups (Table 3). The mean change in microhardness after demineralization compared with baseline (*P* = 0.87) and after remineralization compared with after demineralization (*P* = 0.178) was not significantly different in comparison of coconut milk and control groups (Table 3). Although the decrease from baseline to post-demineralization in the coconut milk group did not reach statistical significance (*P* = 0.110), likely due to the combination of moderate sample size (*n* = 10) and inter-specimen variability (standard deviation ~ 50 units), the overall decrease across all 30 specimens after acidic challenge was significant (*P* = 0.016), confirming the effectiveness of the demineralization protocol.

**Table 3:** Comparison of mean change in enamel microhardness (kgf/mm²) between groups

Comparison	Soy milk	Coconut milk	Control	*P* value (vs. control)
After demineralization – Baseline	-25.8 (91.7)	-44.4 (79.2)	-36.7 (123.4)	0.825 (soy); 0.870 (coconut)
After remineralization – After demineralization	66.2 (111.5)	87.5 (131.2)	19.9 (74.3)	0.289 (soy); 0.178 (coconut)

Values are mean (SD). No significant differences were found between either plant milk and control

## Discussion

This study assessed the effects of soy milk and coconut milk on primary molar enamel microhardness after an acidic challenge. The results showed that the enamel microhardness significantly decreased after the acidic challenge. The enamel microhardness significantly increased after immersion in coconut milk compared with the value after acidic challenge, and even exceeded the baseline value. The first null hypothesis was rejected; soy milk restored enamel microhardness to baseline without significant change. The second and third null hypotheses were accepted, as no significant microhardness differences occurred between coconut milk, soy milk, and artificial saliva groups after intervention.

The enamel microhardness in the control group increased after immersion in artificial saliva but did not reach the baseline level, and this change was not significant. Balakrishnan et al. [16] evaluated the efficacy of two different concentrations of coconut milk and lyophilized coconut extract for remineralization of demineralized dentin and observed that higher concentrations resulted in a significantly higher microhardness, pointing to the remineralizing capacity of coconut products. However, that study used higher concentrations and focused on dentin, whereas our study used commercially available coconut milk on primary enamel, which may explain differences in the magnitude of effect.

Researchers [17] measured the amounts of calcium and phosphate deposited on the enamel surface after immersion in coconut milk, coconut juice, and coconut virgin oil by SEM images. They induced enamel lesions by an acidic challenge and found that all three tested products had remineralizing capacity. In the current study, coconut milk increased enamel microhardness but this increase was not significant compared to the control group. Abd-Elmonsif et al. [14] compared the remineralization potential of cow milk and three plant-based milks namely almond, soy, and barley milks. A key methodological difference is that Abd-Elmonsif used Coca-Cola for demineralization and electron microscopy for assessment, whereas we used citric acid and Vickers hardness testing. Furthermore, their control group was distilled water, which lacks any remineralizing potential, whereas our control was artificial saliva – a more clinically relevant but also more conservative comparator.

They compared the effects of different milks on demineralized enamel and observed that immersion in almond milk had the best remineralizing effect, followed by the cow milk, barley, and soy milk. Also, the morphological remineralization effect shown by electron microscopy indicated that almond milk resulted in a more dispersed remineralization pattern, followed by barley and cow milk. Soy milk resulted in lower mineralized deposits, which was consistent with the present findings. They reported that frequency of immersion in milk, duration of exposure to milk, and acidic challenge are important factors affecting the morphological changes of the enamel surface.

Metly et al. [18]. concluded that penetration of calcium and phosphate ions to restore enamel is limited in pasteurized milk because most of the calcium and phosphate ions are bound to casein groups in pasteurized milk. Soy milk does not contain casein protein; thus, the penetration of Ca^2+^ and phosphate ions into the enamel structure is not affected by milk protein bonds. In the study by Metly et al. [18], both cow milk and soy milk had a remineralizing effect and there was no significant difference between them in this respect. In the present study, although soy milk did not significantly affect enamel microhardness, the microhardness values generally increased to levels higher than baseline, which was consistent with the findings of Metly et al. [18]. Widanti et al. [12] compared the effects on microhardness recovery of cow milk and soy milk on demineralized enamel following an acidic challenge with orange juice. They found that both cow milk and soy milk had cariostatic effects, but soy milk was weaker than cow milk, and none of them could increase the enamel microhardness level above the baseline level. In the present study, immersion in soy milk and coconut milk increased the surface microhardness of the specimens to the baseline level or above it. It should be noted that Widanti et al. [12] used water as the control group; while, artificial saliva was used as the control group in the present study, which can better simulate the oral environment. Abd-Elmonsif et al. [14] compared the effects on microhardness recovery of cow milk and several plant-based milks after an acidic challenge using Coca-Cola. They compared the effects of different milks on demineralized enamel and observed that immersion in soy milk, like immersion in cow milk, increased the P and Ca contents and decreased C, indicating that soy milk had a remineralizing effect, but was less potent than cow milk. It should be noted that electron microscopy was used to examine the surface microhardness of enamel in their study, which has a very high accuracy, and the control group was distilled water. Widyaningtyas et al. [19] concluded that immersion of enamel specimens in soy milk decreased the microporosities of demineralized enamel caused by acid etching. In the present study, although the microhardness of enamel increased after exposure to soy milk and exceeded the baseline value, this increase was not statistically significant. Shen et al. [20] in their clinical trial showed a reduction in depth of lesions and an increase in the salivary mineral content in patients after consumption of cow milk, indicating its remineralizing effect. The discrepancy between Shen’s clinical trial finding that soy milk increased lesion depth and our finding —showing no adverse effect on enamel microhardness—may be explained by the presence of added sugars in the soy milk used in their study, whereas our soy milk contained no added sugars. This highlights the critical importance of checking product formulations.

Also, consumption of soy milk increased the depth of lesions and decreased the salivary mineral content, pointing to its cariogenic effect. However, the present study found no effect of soy milk on enamel microhardness, which may be due to the fact that the soy milk used in their study contained sugar as sweetener, which may be responsible for the observed difference in the results. Lee et al. [21] evaluated the cariogenic potential of various almond milk beverages including bovine milk, sugar-free and sweetened almond milk, and soy milk and reported considerable amounts of Streptococcus mutans biofilm accumulated in soy milk and almond milk groups, pointing to acid production due to sugar fermentation, which was confirmed by a pH analysis. They reported that both soy milk and almond milk was potentially cariogenic but almond milk was not as cariogenic as the soy milk. Huang et al. [6] stated that soy milk, almond products, especially raw almonds, cashew products, and coconut products have a greater potential to cause demineralization and carious lesions than cow milk; while, this effect was not seen in the present study. It should be noted that they used the bacterial biofilm assay in their study. Therefore, methodological differences may explain the conflicting results.

Although both plant milks increased enamel microhardness above post-demineralization levels, only coconut milk achieved a statistically significant increase. Several factors may explain this difference. First, despite soy milk having higher calcium content (120 mg/100 mL) than coconut milk (18 mg/100 mL), coconut milk contains lauric acid – a medium-chain saturated fatty acid also found in breast milk – which may enhance ion penetration into the enamel subsurface. Second, the higher fat content of coconut milk (17 g/100 mL vs. 2 g/100 mL in soy milk) may create a more occlusive film on the enamel surface, potentially reducing further mineral loss and creating a favorable microenvironment for ion exchange. Third, the direct comparison between the two plant milks showed no statistically significant difference (*P* = 0.412), suggesting that the observed within-group significance for coconut milk may be influenced by baseline variability and sample size rather than a true biological superiority. Future studies with larger sample sizes and direct head-to-head designs are needed to determine whether coconut milk truly offers greater remineralization potential than soy milk.

The assessment of surface hardness and structural integrity under chemical or mechanical stress is a common theme across multiple material science and dental studies [22–26]. This methodological approach supports the methodology of the present study for evaluating the influence of plant-based milks, such as coconut and soy milk, on the microhardness of acid-softened primary enamel. Microhardness is a reliable parameter for evaluating the surface resistance of both biomaterials and dental hard tissues [27–30].

In today’s world, the importance of including milk and its derivatives in the daily diet is highly emphasized. They not only neutralize the harmful effects of a diet rich in cariogenic substances, but also prevent other general health problems such as problems related to calcium excretion. Considering this fact, dentists should encourage their patients, especially those suffering from dental erosion, to include dairy milk or non-dairy milks with cariostatic properties in their diet [31]. 

The present study had an in vitro design and thus could not perfectly simulate the complex intraoral environment and the role of microorganisms. Thus, in vivo studies are recommended to obtain more generalizable findings. Also, future studies should include other types of plant-based milks and use bacterial biofilm to assess their cariogenic/cariostatic effects. Moreover, the effect of a combination of bovine and plant-based milks on enamel microhardness should be investigated in future studies.

### Clinical implications

While microhardness recovery in vitro suggests potential benefit, extrapolation to the clinical setting requires caution. Natural saliva contains enzymes (e.g., amylase, lysozyme, lactoferrin), buffering systems (bicarbonate, phosphate, urea), antimicrobial proteins, and a continuous flow that artificial saliva cannot fully replicate. Furthermore, the presence of a salivary pellicle, variable pH, and intermittent acid challenges in the oral environment may substantially alter the effects observed in this simplified model.

### Study limitations

This study has several limitations that should be acknowledged. First, the in vitro design cannot simulate the complex intraoral environment, including salivary flow, pellicle formation, buffering capacity, and microbial biofilm. Second, the 25-hour continuous acid challenge at pH 3 is considerably more aggressive than physiological acid exposures, which are typically intermittent and partially buffered by saliva. Third, cariogenicity involves bacterial metabolism and acid production from fermentable sugars – neither was assessed here; therefore, conclusions about cariogenicity cannot be drawn from microhardness alone. Fourth, the absence of pH cycling (alternating acid and remineralization phases) limits the clinical relevance. Fifth, we did not measure Ca/P ratios or subsurface lesion profiles, so we cannot confirm true remineralization rather than surface hardening. Sixth, the short 7-day duration and use of only one brand per milk type limit generalizability. Seventh, the use of artificial saliva as a storage medium between milk immersions may have contributed to the observed microhardness increases, making it difficult to isolate the specific effect of each milk.

## Conclusion

Under in vitro settings, the effects on microhardness recovery of coconut milk and soy milk were not significantly different from that of artificial saliva; however, they increased the microhardness (significantly by coconut milk and insignificantly by soy milk) after an acidic challenge, and therefore, they were not cariogenic. This study assessed the effects of soy milk and coconut milk on primary molar enamel microhardness after an acidic challenge. The results showed that the enamel microhardness significantly decreased after the acidic challenge across all groups. Coconut milk immersion significantly increased microhardness compared to post-demineralization values, while soy milk and artificial saliva showed non-significant increases. Between-group comparisons revealed no significant difference between either plant milk and artificial saliva.

## Data Availability

The data used to support the findings of this study were supplied by corresponding author under license and data will be available on request. Requests for access to these data should be made to corresponding author.

## References

[1] Sabel N. Enamel of primary teeth—morphological and chemical aspects. Swed Dent J Suppl. 2012;(222):1–77. 22515039

[2] Petersen P PE. The World Oral Health Report 2003: continuous improvement of oral health in the 21st century–the approach of the WHO Global Oral Health Program. Community Dent Oral Epidemiol. 2003;31(Suppl 1):3–23. 10.1046/j..2003.com122.x 15015736

[3] Bossù M, Saccucci M, Salucci A, Di Giorgio G, Bruni E, Uccelletti D, et al. Enamel remineralization and repair results of biomimetic hydroxyapatite toothpaste on deciduous teeth: An effective option to fluoride toothpaste. J Nanobiotechnol. 2019;17(1):1–13. 10.1186/s12951-019-0454-6 PMC6346538 30683113

[4] Kanzow P, Wegehaupt FJ, Attin T, Wiegand A. Etiology and pathogenesis of dental erosion. Quintessence Int. 2016;47(4):275–8. 10.3290/j.qi.a35625 27022647

[5] Johansson AK, Sorvari R, Birkhed D, Meurman JH. Dental erosion in deciduous teeth—an in vivo and in vitro study. J Dent. 2001;29(5):333–40. 10.1016/s0300-5712(01)00029-x 11472805

[6] Huang Y, Thompson T, Wang Y, Yu Q, Zhu L, Xu X, et al. Analysis of cariogenic potential of alternative milk beverages by in vitro Streptococcus mutans biofilm model and ex vivo caries model. Arch Oral Biol. 2019;105:52–8. 10.1016/j.archoralbio.2019.05.033 PMC6629491 31276938

[7] Sethi S, Tyagi SK, Anurag RK. Plant-based milk alternatives an emerging segment of functional beverages: a review. Journal of food science and technology. 2016;53(9):3408–23. 10.1007/s13197-016-2328-3 PMC5069255 27777447

[8] Vanga SK, Raghavan V. How well do plant based alternatives fare nutritionally compared to cow’s milk?. Journal of food science and technology. 2018;55(1):10–20. 10.1007/s13197-017-2915-y PMC5756203 29358791

[9] El Deeb M, Abdel Moneim RA. Remineralization potential of lactose-free and plant-based milk on enamel surface of human teeth subjected to energy drinks. EDJ. 2018;64(4):3439–52.

[10] Sumner O, Burbridge L. Plant-based milks: the dental perspective. British Dental Journal. 2020:1–7. 10.1038/s41415-020-2058-9. 32918058

[11] Collard KM, McCormick DP. A nutritional comparison of cow’s milk and alternative milk products. Acad Pediatr. 2021;21(6):1067–9. 10.1016/j.acap.2020.12.007 33373745

[12] Widanti H, Herda E, Damiyanti M. Effect of cow and soy milk on enamel hardness of immersed teeth. J Phys. 2017;884(1):012006.

[13] Shetty V, Bhandary S, Vakil I. Remineralizing potential of milk and GC Tooth Mousse on demineralized human enamel: An in vitro comparative evaluation. J Health Allied Sci NU. 2022;13(01):107–13.

[14] Abd-Elmonsif NM, El-Zainy A, Medhat MM, Abd-elhamid MA. Comparative study of the possible effect of bovine and some plant-based milk on cola-induced enamel erosion on extracted human mandibular first premolar. Futur Dent J. 2017;5(3):22–7.

[15] Ghavri T, Pathak A, Bajwa NK. Comparative evaluation of the effect of different beverages on the surface roughness and microhardness of human enamel surface: An in vitro study. Int J Clin Pediatr Dent. 2021;14(5):657–61. 10.5005/jp-journals-10005-2011 PMC8645618 34934279

[16] Balakrishnan N, Subramanian AK, Eeswaramoorthy R, Kandasamy D. Enamel remineralization efficacy of coconut milk and lyophilized coconut extract in different concentrations on demineralized enamel surfaces: An in vitro study. Cureus. 2023;15(9):e44712. 10.7759/cureus.44712 PMC10552678 37809240

[17] Balakrishnan N, Subramanian AK, Eeswaramoorthy R, Kandasamy D, Kumar A, Eswaramoorthy R. Enamel Remineralization Efficacy of Coconut Milk and Lyophilized Coconut Extract in Different Concentrations on Demineralized Enamel Surfaces: An In-Vitro Study. Cureus. 2023;15(9). 10.7759/cureus.44712 PMC10552678 37809240

[18] Metly A, Sumantri D, Oenzil F. The effect of pasteurized milk and pure soy milk on enamel remineralization. Padjadjaran J Dent. 2019;31(3):202–7.

[19] Rahayu YC. Analisis Peningkatan Remineralisasi Enamel Gigi setelah Direndam dalam Susu Kedelai Murni (Glycine max (L.) Merill) Menggunakan Scanning Electron Microscope (SEM)(The Analysis of Enamel Remineralization Increase in Pure Soy Milk (Glycine max (L.) Merill). Pustaka Kesehatan. 2014;2(2):258–62.

[20] Shen P, Walker GD, Yuan Y, Reynolds C, Stanton DP, Fernando JR, et al. Effects of soy and bovine milk beverages on enamel mineral content in a randomized, double-blind in situ clinical study. J Dent. 2019;88:103160. 10.1016/j.jdent.2019.06.007 31238081

[21] Lee J, Townsend JA, Thompson T, Garitty T, De A, Yu Q, et al. Analysis of the cariogenic potential of various almond milk beverages using a Streptococcus mutans biofilm model in vitro. Caries Res. 2018;52(1–2):51–7. 10.1159/000479936 PMC5828958 29241218

[22] Lin H, Yin X, Wang Z, Xu J, Wang Y, Cheng S, et al. Friction-Induced PTFE Coating on Dental Restorative Resin with Ultralow Friction and Wear. Langmuir. 2025;41(33):22501–6. 10.1021/acs.langmuir.5c03007 40802483

[23] Li C, Wada T, Tsuchida Y, Hayashi K, Tanabe G, Ueno T, et al. Optimizing additively manufactured mouthguards: An evaluation of multi-layer materials for improved shock absorption and durability compared to conventionally fabricated samples. Int J Bioprint. 2024;10(3):2469.

[24] Xu D, Zhang S, Qin Y. Study of the Micromechanical Properties and Dissolution Characteristics of Porous Coral Reef Limestone. J Geophys Res Solid Earth. 2024;129(11):e2024JB029131.

[25] Geng T, Ding L, Liu M, Zou X, Gu Z, Lin H, et al. Preservation of extracellular vesicles for drug delivery: A comparative evaluation of storage buffers. J Drug Deliv Sci Technol. 2025;107:106850.

[26] Pan Y, Du Z, Ma Y, Chen C, He S, Zhang M, et al. Suppression effect of folate on poly- and perfluoroalkyl substance-induced alterations in lipids and the atherogenic index of plasma in adolescents. Lipids Health Dis. 2025;24(1):213. 10.1186/s12944-025-02638-y PMC12153114 40500729

[27] Khandan A, Abdellahi M, Ozada N, Ghayour H. Study of the bioactivity, wettability and hardness behaviour of the bovine hydroxyapatite-diopside bio-nanocomposite coating. J Taiwan Inst Chem Eng. 2016;60:538–46.

[28] Jamnezhad S, Asefnejad A, Motififard M, Yazdekhasti H, Kolooshani A, Saber-Samandari S, et al. Development and investigation of novel alginate-hyaluronic acid bone fillers using freeze drying technique for orthopedic field. Nanomed Res J. 2020;5(4):306–15.

[29] Kazemi Roudsari P, Attarzadeh H. Effect of fresh orange juice and industrial orange juice on enamel microhardness of primary teeth: an in-vitro study. Front Dent. 2026;23:9. 10.18502/fid.v23i9.21275. PMC13428511 42542871

[30] Jensdottir T, Bardow A, Holbrook P. Properties and modification of soft drinks in relation to their erosive potential in vitro. J dent. 2005;33(7):569–75. 10.1016/j.jdent.2004.12.002 16005796

[31] Oliveira TA, Scaramucci T, Braga SR, Sobral MAP. The effect of milk ingestion after an orange juice in the superficial microhardness of human enamel: An in vitro study. RPG Rev Pós-Grad. 2012;19(3):89–94.

